# Molecular ingredients of an immunogen for long-lasting IgG

**DOI:** 10.3389/fimmu.2025.1639371

**Published:** 2025-08-19

**Authors:** Sneh Lata Gupta, Alexander R. Meyer, Erika Kay-Tsumagari, Wei Cheng

**Affiliations:** ^1^ Department of Pharmaceutical Sciences, University of Michigan, Ann Arbor, MI, United States; ^2^ Department of Biological Chemistry, University of Michigan Medical School, Ann Arbor, MI, United States

**Keywords:** virus, vaccine, antibody, IgG, persistence, durability

## Abstract

The durability of vaccine-induced protection is a critical parameter in assessing the overall quality and long-term effectiveness of a vaccine. While the lifelong immunity conferred by certain vaccines is well recognized, the molecular components that underpin such long-lasting protection remain poorly understood. This knowledge gap is further complicated by the frequent inclusion of adjuvant formulations in licensed vaccines, the mechanisms of which are often multifaceted and not fully elucidated. In this review, drawing upon the portfolio of FDA-approved antiviral vaccines and incorporating insights from our own published studies in rodents, we propose that a virus-like structure - devoid of any engineered adjuvants - is all that is needed for a long-lasting IgG response in both mice and humans. This structure comprises two essential features: (1) the oriented display of viral surface protein antigens on a virus-sized scaffold, and (2) internal nucleic acids with native phosphodiester backbones. In fact, several inactivated virus vaccines that conform to this architecture have demonstrated effective and durable protection in human populations without the need for engineered adjuvants. Clarifying these structural and molecular determinants of viral immunogenicity may reduce the empirical nature of vaccine development, enable the rational design of next-generation self-adjuvanting antiviral vaccines, and inspire novel applications in noncommunicable diseases.

## Introduction

Vaccination is a concept that can be broadly applied to the prevention and treatment of diseases, including but not limited to infectious diseases, cancer and neurodegenerative diseases. Vaccination has controlled more than a dozen human diseases, with the COVID-19 pandemic as the most recent example. Moreover, vaccines have shown promise in cancer treatment (1–3) and tumor prevention (4). Clinical trials of vaccination for type 1 and type 2 diabetes (5, 6), hypertension (7) and Alzheimer’s disease (8, 9) have been conducted in several countries. These studies suggest a much broader applicability of vaccination in global public health including noncommunicable diseases (10). In fact, the list of diseases that are in need of vaccines far exceeds the list of diseases for which licensed vaccines are available (11).

However, at a mechanistic level, our understanding of the vaccine efficacy together with the durability of protection remains limited. For example, as of current, the rules that govern the durability of vaccine protection are yet to be defined (12). Some vaccines, such as those against measles, mumps, and rubella, generate antibodies (Abs) whose plasma concentration half-lives span the lifetime of an individual (13); others, such as SARS-CoV-2 mRNA vaccines, generate Abs with half-lives of months (14) and do not establish long-lived plasma cells in the bone marrow (15). This wide discrepancy highlights a gap in our knowledge of B cell responses to antigens (Ags), as different vaccine platforms fail to induce a durable Ab response consistently and reproducibly. This gap in our knowledge, if unfilled, will continue to hamper our ability to develop effective and durable vaccine formulations to combat various diseases.

Along this vein of research, excellent work has been put forward by Slifka and coworkers regarding the durability of Ab responses induced by vaccines (16). The current manuscript is not meant to repeat the lines of work published previously, but rather to focus on aspects of vaccines that have not been heavily examined. These insights, combined with our own recent work in rodents, lead us to propose our views of the structural and molecular components which trigger long-lasting plasma Ab responses.

In organizing this review, we have chosen to examine antiviral vaccines that have been licensed in the US in chronological order. To focus our discussion, we have mostly limited our study to live attenuated vaccines, inactivated vaccines or virus-like particles, because we feel that at a mechanistic level, they belong to the shared category of vaccines that is based on the biochemical and biophysical structures of virions. Therefore, they would share common aspects of immune system activation and the maintenance of an immune response. In chronological order of their approval in the US, these vaccines are listed in **Table 1**.

**Table 1:** List of US FDA approved live attenuated, inactivated or virus-like particle vaccines in chronological order.

Year	Vaccine	Genetic material in the vaccine	Vaccine composition and doses			Persistence of sera Ab
			Vaccine form	Additional adjuvants?	# of doses	
1931	Smallpox vaccine	dsDNA, ~ 190 kb	Live virus, unattenuated	No	One	Potentially lifelong
1938	Yellow fever vaccine	(+)ssRNA, ~ 11 kb	Live attenuated virus	No	One	Long but pronounced waning in children
1945	Influenza vaccine	(-)ssRNA, ~ 14 kb total^(A)^	Whole inactivated virus	Variable depending on the vaccine form	One or two depending on patient history	Variable depending on the vaccine form
			Split virus			
			Live attenuated virus, 2003			
1955	Polio vaccine	(+)ssRNA, ~ 7.4 kb	Inactivated virus	No	Four	Potentially lifelong
			Live attenuated virus, 1961	No	Three or four depending on countries	
1963	Measles vaccine	(-)ssRNA, ~ 16 kb	Live attenuated virus	No	Two	Potentially lifelong
1967	Mumps vaccine	(-)ssRNA, ~ 15 kb	Live attenuated virus	No	Two	Potentially lifelong
1969	Rubella vaccine	(+)ssRNA, ~ 9.8 kb	Live attenuated virus	No	Two	Potentially lifelong
1980	Rabies vaccine	(-)ssRNA, ~ 12 kb	Inactivated virus	No	Three^(B)^	>10 years
1981	Hepatitis B vaccine	None	Subvirion particles	Alum	Three	>30 years
1992	Japanese encephalitis vaccine	(+)ssRNA, ~ 11 kb	Inactivated virus (JE-VAX)	No	Three	>2 years
			Inactivated virus (IXIARO) (2009)	Alum	Three	>6 years
1995	Varicella (chickenpox) vaccine	dsDNA, ~ 125 kb	Live attenuated virus	No	Two	>20 years
1995	Hepatitis A vaccine	(+)ssRNA, ~ 7.5 kb	Inactivated virus	Alum	One or two^(C)^	>25 years
2006	Human Papillomavirus (HPV) Vaccine	None	Virus-like particles	Alum or Alum+MPLA	One or two^(D)^	>10 years
2006	Rotavirus Vaccine	dsRNA, ~ 19 kb total	Live attenuated virus	No	Three	Variable depending on setting
2006	Zoster (Shingles) Vaccine	dsDNA, ~ 125 kb for live attenuated	Live attenuated virus	No	One	Protection wanes substantially within 10 years
		None	Subunit (2017)	AS01_B_	Two	>10 years
2011	Adenovirus vaccine	dsDNA, ~ 36 kb (Ad4), ~ 35 kb (Ad7)	Live virus, unattenuated	No	One	>6 years
2019	Dengue vaccine	(+)ssRNA, ~ 11 kb	Live chimeric virus	No	Three	>3 years
2023	Chikungunya vaccine	(+)ssRNA, ~ 12 kb; (E)	Live attenuated virus (IXCHIQ)	No	One	>1 year
			Virus-like particles (VIMKUNYA) (2025)	Alum	One	>2 years

(A) The split virus most likely retains viral RNA because formaldehyde can trigger the crosslinking between proteins and RNA, which is a procedure before virion disruption by detergent.

(B) This for prophylaxis. For post-exposure situation varies.

(C) Besides standard 2-dose vaccination, World Health Organization also included single-dose vaccination in its position paper as of 2022 (242).

(D) Besides standard 2-dose vaccination, World Health Organization also included single-dose vaccination in its position paper as of 2022 (243).

(E) Chikungunya virus-like particle vaccine VIMKUNYA likely contains cellular RNA from HEK293 cells.

Discussion of other vaccine platforms such as mRNA vaccines is not the focus of the current study, mainly due to the distinct forms of immunogen presentation utilized by these platforms as compared to the above category. Because the biophysical form of an Ag is absolutely critical for B cell Ab responses (17, 18), the mRNA vaccines likely activate B cells differently than particulate vaccines, and the development and maintenance of the responses may also differ as a result, especially regarding mechanisms at the molecular and cellular level. In our discussion, we pay special attention to the use of any adjuvants in vaccine formulations. Because these adjuvants usually act through mechanisms that have not been well defined, they make the resulting immune responses more complex to interpret. In our discussion of mechanisms of vaccine-induced protection, we focus on plasma antibody responses because of the relative abundance of literature data to support this discussion. Alternative mechanisms such as anamnestic responses mediated by memory B or T cells are likely to be important as well but will not be the focus of our discussion. In structuring the current manuscript, we will first examine these antiviral vaccines following the order in **Table 1**, review their compositions in detail, and focus on the durability of the plasma antibody responses they elicit. We will then incorporate our own published studies in rodents and conclude by drawing implications from this collective knowledge.

## Smallpox vaccine

The smallpox vaccine received approval from the FDA in 1931. Historically, it was Dr. Edward Jenner who inoculated an 8-year-old boy in 1796 using matter from a cowpox sore; the boy then remained healthy after challenge with matter from a human smallpox sore (19). This pioneering practice laid the foundation for contemporary immunology. The Dryvax formulation for smallpox vaccine initially approved by the FDA is a live-virus preparation of vaccinia virus harvested from calf lymph. As pointed out by Slifka and Amanna (16), this virus has not been specifically attenuated. This formulation contains the lyophilized virus prep and perhaps trace amounts of antibiotics carried over from the vaccine processing process. After the reconstitution of the lyophilized vaccine, it was tested that no more than 200 per ml of viable bacterial organisms were present in the final product. This is an important quality test because this vaccine is prepared from live stocks and bacterial contamination would be a concern. Aside from the sterility required for parenteral applications of a dosage form such as Dryvax, bacteria, even trace amounts of carryover from the original live materials, could act as natural adjuvants for this vaccine, which has to be considered regarding the mechanisms of immune system activation by the vaccine. After reconstitution, this vaccine contains approximately 100 million infectious vaccinia viruses per ml of the suspension, which converts to a molar concentration of 0.17 pM for the infectious virions. This vaccine is administered percutaneously via punctures into the superficial layer of the skin. The efficacy of this vaccine is showcased by the remarkable eradication of smallpox worldwide by 1980 (20). As demonstrated by Hammarlund et al. (21), the vaccinia-specific Ab in the serum of vaccinees displayed remarkable stability over decades in a cross-sectional analysis among 306 vaccinees. In a separate independent study of 27 subjects, the vaccinia-specific memory B cells were shown to be stably maintained for >50 years at a frequency of ~0.1% of total circulating IgG^+^ B cells (22). Furthermore, the half-life of vaccinia-specific Ab decay has been estimated to be 92 years in a longitudinal analysis of 45 subjects who were either vaccinated with or naturally exposed to vaccinia virus (13). Therefore, even though epidemiology data suggest that there is waning immunity after the primary one-dose vaccination of the smallpox vaccine (23), this waning immunity may not be significant over the typical human lifespan.

## Yellow fever vaccine

The second vaccine in **Table 1** is the yellow fever vaccine approved in 1938. The YF-VAX formulation of yellow fever vaccine is a live attenuated 17D-204 strain of the yellow fever virus cultured from chicken embryos. This formulation contains the lyophilized virus prep, together with sorbitol and gelatin as stabilizers. It has no preservatives or other adjuvants. The durability of immune protection offered by a single dose of this attenuated virus has been controversial especially in recent years (24). It has been reported that ~21% of adults may substantially lose their neutralizing Abs at ~ 10 years post vaccination (25). The situation is even worse in children. In both Africa and South America, a significant loss of neutralizing Ab titers in children has been reported just after several years post vaccination (26, 27). These data point to the complexity behind the maintenance of serum Abs after vaccination in human populations, with the mechanisms of Ab maintenance and its age dependence still to be investigated. Early studies on this attenuated vaccine clearly indicated the pivotal role of Abs in mediating immune protection (28). Memory B cell responses have also been characterized recently up to one year after vaccination (29). However, the lifespan of these memory B cells remains to be studied. Furthermore, Pulendran et al. have used a systems biology approach to understand the mechanisms of immune system activation after administration of the yellow fever vaccine. They revealed that cell-intrinsic stress responses may be critical in immune system activation (30). Lastly, despite being a highly efficacious vaccine overall, the yellow fever vaccine suffers from infrequent but severe neurotropic adverse effects linked to viral replication, which have been observed in both infants and adults (28). Partly because of these severe adverse events, the yellow fever vaccine has not become a vaccine of recommendation for the general public (31), but one that is recommended for travelers in the US.

## Influenza vaccine

**Figure 1:**
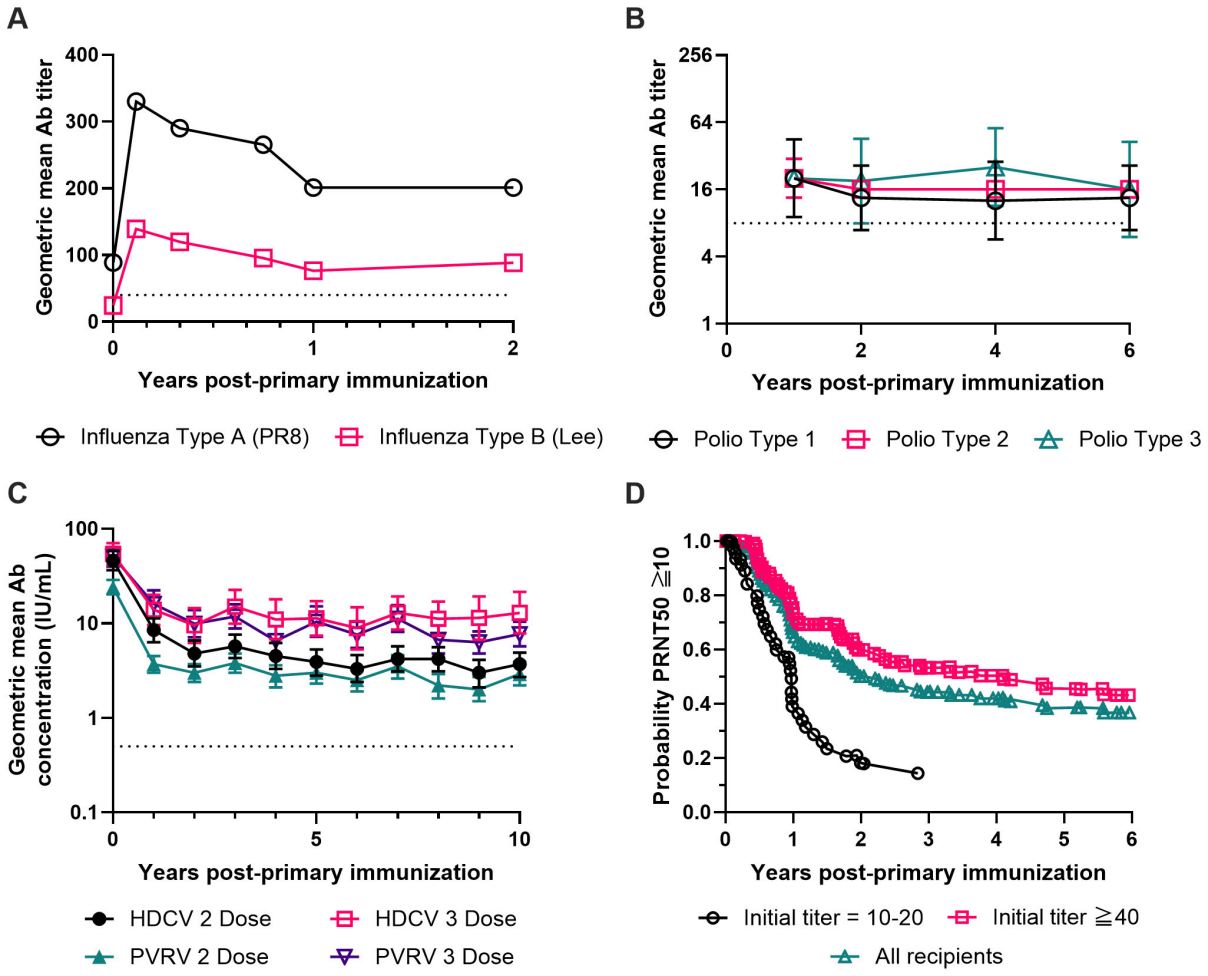
Long-term antibody responses after completion of a primary immunization series for four inactivated virus vaccines without exogenous or engineered adjuvants. **(A)** Geometric mean antibody titers against influenza virus Type A strain PR8 (black circles) and influenza virus Type B strain Lee (red squares) measured for 2 years after completing a primary immunization series. Subjects (n=45) were immunized with a single dose of a formalin-inactivated influenza virus vaccine containing influenza strains PR8 (Type A), FM1 (Type A’) and Lee (Type B) in aqueous solution. The dashed line at a titer of 40 marks the threshold hemagglutination inhibition titer which indicates seroconversion (244). Data were replotted from the two left panels of Figure 1 from Salk et al. (34). **(B)** Geometric mean antibody titers against poliovirus Type 1 (black circles), Type 2 (red squares) and Type 3 (green triangles) measured for 6 years after completing a primary immunization series. Seronegative children (n=4) received three doses of a formalin-inactivated poliomyelitis virus vaccine at two-week intervals containing poliovirus strains Mahoney (Type 1), MEF-1 (Type 2), and Saukett (Type 3). The dashed line at a titer of 8 marks the minimum titer used by the CDC to indicate protective levels of antibody against poliovirus (245). The figure was prepared using the geometric mean of the data shown in Figure 14 of Salk (50) with error bars representing the geometric standard deviation. **(C)** Geometric mean neutralizing antibody concentrations against rabies virus measured for 10 years after completing a primary immunization series. Subjects (n=312) received either a 2 dose or 3 dose primary series of the β-propiolactone-inactivated human diploid cell rabies vaccine (HDCV, black circles and red squares) or the β-propiolactone-inactivated purified Vero cell rabies vaccine (PVRV, green triangles and purple triangles). The dashed line at 0.5 IU/mL marks the WHO-recommended concentration which indicates seroconversion (246). The figure was prepared using the data provided in Table 3 from Strady et al. (65) with error bars representing the 95% confidence interval of the geometric mean. **(D)** Kaplan-Meier estimation of how long the 50% plaque reduction neutralization (PRNT50) titer against Japanese encephalitis (JE) virus remains above or equal to 10 over a period of 6 years after completing a primary immunization series. Subjects (n=293) received a 3 dose primary series of a formalin-inactivated JE virus vaccine from one of 22 different vaccine lots. Vaccine recipients were then screened for initial JE PRNT50 titers within 56 days of completing the primary vaccine series. Vaccine responders (n=269) were divided into three groups for survival analysis based on their initial PRNT50 titer values: Initial PRNT50 titer = 10-20 (black circles), Initial PRNT50 titer ≧ 40 (red squares), or the weighted average of all vaccine responders (green triangles). Data were replotted from Figure 2 of Reisler et al. (82).

The first influenza vaccine was developed by Thomas Francis Jr. and Jonas Salk at the University of Michigan (32, 33), and it was licensed for civilian use in 1945. In this initial development, the viruses were cultured in chick embryos, harvested and inactivated with a 1:2000 dilution of formalin. Phenyl mercuric nitrate was also added at a 1:100,000 dilution for bacteriostatic purposes. Despite having no other adjuvants in this formulation, this aqueous vaccine was shown to elicit durable Ab titers in human subjects up to two years post vaccination (**Figure 1A**), although the same vaccine adjuvanted in an emulsion formulation with light mineral oil elicited a much higher magnitude of Abs (34). The technology of influenza vaccine manufacturing has evolved over the years since then, of which Slifka and Amanna have given an excellent review (16). They pointed out the important differences in Ab durability between whole inactivated viruses and the so-called split-virions, which are virion structures disrupted by treatment with detergent such as sodium deoxycholate. Both the vaccine efficacy and anti-hemagglutination titer waned more quickly for split virions, a trend which was reported in 1977 in an independent study conducted by Cate and coworkers (35). The integrity of the virion structure in a vaccine formulation is an important factor influencing Ab durability and must be considered when the durability of vaccine protection is of concern. This becomes an urgent issue, especially in light of the extensive literature around the world that has documented the short-lived protection after influenza vaccination (36–40).

In addition to inactivated influenza vaccines, a live attenuated influenza vaccine, FluMist (41), was licensed in 2003. This live attenuated vaccine contains three circulating viral strains without additional adjuvants or preservatives and is administered through an intranasal spray. FluMist has been shown to be safe and effective in healthy, working adults (42). Moreover, it showed a very good efficacy of 93% in a randomized, double-blind, placebo-controlled trial that involved 1314 children 15 through 71 months of age (43). However, in two independent clinical studies, Barría et al. showed that FluMist has only a 9% seroconversion rate in adults (44), and Couch et al. showed that FluMist was a poor inducer of serum Abs compared to inactivated vaccines in healthy adults (45).

## Polio vaccine

Broadly, there are two versions of polio vaccine in the world: inactivated polio vaccine (IPV) and oral polio vaccine (OPV), the latter being a live attenuated polio virus. Due to the low but definitive risk of poliomyelitis associated with OPV (46, 47), the US has stopped using OPV since 2000 (48) and therefore we will focus on IPV in terms of its formulation and durability of protection. IPV was developed by Jonas E. Salk in the early 1950s (49). Although the vaccine production procedure has been much improved nowadays as techniques for purification of viruses become more mature, the key step in this vaccine production remains unchanged: the inactivation of viruses with 1:4000 formaldehyde. No other adjuvants were included. A single dose of the IPV already confers protective immunity in humans, but the vaccine efficacy increases progressively with the number of doses (50). In fact, current practice for children in the US is 4 doses of IPV vaccine at 2, 4, 6–18 months and 4–6 years of age (48). Early studies by Salk showed that poliovirus-specific Abs were persistent in children for years after three doses of IPV (50) (**Figure 1B**), which is very impressive given the fact that no adjuvants other than the inactivated viruses are present in this formulation. Moreover, in a clinical study that involved 53 term and 13 preterm infants, nasopharyngeal IgA antibodies can be detected in 43% to 91% of the infants depending on age after 3 doses of IPV by subcutaneous injections (51), indicating that IPV not only has a high rate of seroconversion, but also can induce localized mucosal immune responses with a very good probability. Lastly, it should be noted that the stable Ab titers observed from individual patients after the completion of a primary immunization series (**Figure 1B**) has no indication on the time course of Ab titers during the first year. Based on the data from a patient who was monitored more frequently in the same study (50), a substantial decay in Ab titer (over tenfold) could well occur within one year post vaccination. In fact, a multi-phase decay is likely a recurring feature of Ab titer change with time in human vaccination, as revealed from previous modeling (16) and what we shall discuss in this work.

## Measles, mumps and rubella vaccine

The MMR vaccine is a blend of three live attenuated virus vaccines against measles, mumps and rubella. The vaccine product is a sterile lyophilized preparation of three live attenuated viruses without additional adjuvants or preservatives. The efficacy of the MMR vaccine has been among the best of all antiviral vaccines known to date, with the reduction in annual morbidity ≥99% since its introduction (52, 53). In most individuals, neutralizing Abs to measles, mumps and rubella viruses are long-lived and persistent for years after primary vaccination (54–57). However, the waning of Ab titers with time in different populations around the world has been reported for all three viruses even after the second dose of the MMR vaccine (58–62).

## Rabies vaccine

Rabies vaccine is another example of an inactivated virus that is highly effective in both prophylactic and post-exposure settings. The rabies vaccines approved in the US, including IMOVAX by Sanofi Pasteur and RabAvert by Novartis, are suspensions of freeze-dried rabies virus prepared from cell culture and inactivated with β-propiolactone. The formulations contain no preservatives or other adjuvants and are provided for intramuscular injection for both children and adults. For pre-exposure prophylaxis, three 1.0-ml doses are recommended on Days 0, 7, and 21 or 28 (63). In a clinical study conducted in the UK that involved 194 subjects and up to 3 years of follow-up after primary immunization with one, two, or three doses of rabies vaccines derived from human diploid cells, 95% subjects were seroconverted after the first dose (64). However, the virus-specific Ab titer dropped significantly within the first 6 months after a single dose. A later clinical study was conducted in France that involved 312 subjects and 10 years of follow-up after primary immunization with two or three doses of cell culture derived and inactivated rabies vaccines (65). All subjects received one additional booster dose one year after the primary immunization. In this study, both primary vaccination regimes (2 doses and 3 doses) were observed to mediate persistent Ab titers over the 10-year span (**Figure 1C**).

## Hepatitis B vaccine

The first hepatitis B vaccine approved in the US in 1981 was Heptavax-B produced by Merck. This vaccine was prepared by purifying hepatitis B virus (HBV) 22-nm subvirion particles from the plasma of asymptomatic chronic HBV-infected patients followed by inactivation (66) and adjuvanting with alum (67). It was subsequently discovered that the HBV surface Ag (HBsAg) expressed from the yeast *Saccharomyces cerevisiae* is actually assembled into particles that resemble these 22-nm subvirion particles (68). This technique has allowed Merck to develop a 2^nd^-generation hepatitis B vaccine—Recombivax HB—using recombinant DNA technology in conjunction with yeast protein expression (69), therefore bypassing the limited supply restrictions of chronic hepatitis B patients. The same technology was later adopted by GlaxoSmithKline Biologicals, who developed Engerix-B as a hepatitis B vaccine. In both formulations, the purified recombinant HBsAg is adsorbed onto alum as the adjuvant. Specifically for each 10 μg dose of HBsAg, there is 250 μg of aluminum hydroxide for Recombivax HB and 500 μg of aluminum hydroxide for Engerix-B. The persistence of protective immunity induced by different versions of hepatitis B vaccines has been extensively studied in the literature. In a clinical study involving 243 subjects initially immunized with Heptavax-B, the concentration of Abs against HBsAg (anti-HBs) decreased ~ 10-fold during the first 10 years post vaccination, and the level of serum Ab continued to drop over the next 20 years although at a slower pace (70). Among these, 51% of the subjects retained anti-HBs at a level above 10 mIU/ml, which was considered protective. For those subjects with anti-HBs below 10 mIU/ml, they were given a booster dose of Engerix-B, and 88% had an anamnestic response with anti-HBs >10 mIU/ml. Based on these overall results, it was concluded that >90% of subjects showed evidence of protection 30 years later and booster doses are not needed (70). Similar conclusions were also supported by independent studies in Canada (71). Despite the durable protection that has been achieved with hepatitis B vaccines, it is interesting to note that the rapid drop in anti-HBs titer during the first 10 years post vaccination has been reproducibly observed in several independent studies that were carried out in different regions around the world, including Israel, the US, Italy, Taiwan, Thailand, and Gambia (72–77). Lastly, it is worth mentioning the 3^rd^ generation of hepatitis B vaccine, HEPLISAV-B, that was approved by FDA in 2017. This formulation uses HBsAg expressed from the yeast *Ogataea polymorpha* admixed with CpG1018, a 22-mer oligodeoxynucleotide with a phosphorothioate backbone as the adjuvant. In each 0.5-ml dose for intramuscular injection, there are 20 µg of HBsAg and 3000 µg of CpG1018 (78). In a recent observation study involving 147 participants who had chronic kidney disease and received either HEPLISAV-B or Engerix-B, the durability of the protective Ab response (anti-HBs >10 mIU/ml) was quantitatively similar between the two vaccines over three years, although the geometric mean titer of anti-HBs was maintained at higher levels over time following HEPLISAV-B immunization (79).

## Japanese encephalitis vaccine

JE-VAX was the first Japanese encephalitis (JE) vaccine approved in the US in 1992, which was an inactivated JE virus derived from mouse brain. It was provided as a sterile lyophilized vaccine for for subcutaneous injection upon reconstitution. To manufacture JE-VAX, the virus was first inoculated into mice intracerebrally for viral replication and propagation. The infected brain was then harvested and homogenized in phosphate-buffered saline. The homogenate was then centrifuged, and the supernatant was inactivated with formaldehyde. The supernatant containing the inactivated virus was further purified through sucrose gradient ultracentrifugation and lyophilized. The formulation also contained thimerosal as a preservative without other known adjuvants. In a placebo-controlled blinded clinical trial that involved 65,224 children in Thailand (80), two doses of JE-VAX were shown to be 91% effective in reducing encephalitis attack rates among the enrolled subjects over the study period of ~ 2 years. However, the full duration of protection offered by this vaccine was still unknown. In a subsequent study involving US soldiers (81), it was reported that virus-specific serum Ab titers dropped quickly within the first year. The seropositivity rate was 85% at 8 weeks after two doses of the vaccine but dropped to 33% by 26 weeks post vaccination, which could be boosted to 100% within a month after a 3^rd^ booster dose. In a later study involving 293 subjects at the US Army Medical Research Institute of Infectious Diseases who received a three-dose primary series of JE-VAX, seropositivity waned to 50% at 805 days based on quantitative analysis of the PRNT50 data (82) (**Figure 1D**). Lastly, independent studies in Japan reported that protective levels of neutralizing Abs in children were maintained for at least 3–5 years after 3 doses of JE-VAX (83).

Presently, JE-VAX is no longer produced and all remaining doses expired in 2011 (84). It has been replaced by IXIARO, a second-generation JE vaccine that was approved in 2009 by the US FDA. IXIARO is an inactivated JE virus vaccine derived from Vero cells instead of mouse brain and is provided as a sterile suspension for intramuscular injection. Compared to JE-VAX, IXIARO has a more stringent purification procedure for the viruses. The purified viruses are inactivated with formaldehyde and further adsorbed onto alum as the adjuvant. Each 0.5-ml dose of the vaccine contains approximately 6 µg of JE virus proteins and 250 µg of aluminum hydroxide with no added preservatives. The durability of protection offered by IXIARO in human populations has been studied, although not as extensively as the hepatitis B vaccines reviewed above. From this limited number of studies (85–87), it is clear that a two-dose primary immunization followed by a booster one year later offers protection for at least 6 years post vaccination. The titer of virus-specific neutralizing Abs in the serum of participants dropped quickly during the first year after immunization, but this reduction in titer became much slower over the next few years after the booster immunization. A quantitative analysis by Slifka and Amanna suggested that a 3-dose regime of IXIARO could potentially provide lifelong protection against JE (16). Lastly, it is worth noting that while not licensed in the US, other live attenuated vaccines for JE have been available internationally for some time. These include the SA14-14–2 live attenuated vaccine (88), which utilizes the same JE virus strain as IXIARO, as well as the novel chimeric vaccine Imojev. Imojev was constructed using the yellow fever vaccine 17D as the backbone with the insertion of SA-14-14–2 envelope proteins. Clinical trials showed that both live vaccines are safe and effective. Notably, a single dose of Imojev appears to mediate long-lasting protection based on clinical trials conducted in Australia (89) and Thailand (90).

## Varicella (chickenpox) vaccine

VARIVAX manufactured by Merck was approved by the US FDA in 1995 for the prevention of varicella (chickenpox) in children and adults. The vaccine is a live attenuated varicella virus harvested from human diploid cell line MRC-5. VARIVAX contains the lyophilized virus prep without preservatives or other adjuvants. The vaccine is to be reconstituted as a sterile suspension for subcutaneous injection. Two doses of the vaccine appear to mediate durable protection. One clinical study conducted by Merck (91) followed ~ 2,000 children for 10 years who had received either one or two doses of VARIVAX. The incidence of developing varicella showed that a 2-dose regime is threefold more effective than a one-dose regime in preventing varicella. The persistence of virus-specific Abs was studied using ELISA and interestingly, the geometric mean titer of the Abs did not wane with time within the 10-year follow-up for either the one-dose or two-dose groups, with even signs of increase with time, suggesting contributions from exogenous exposure, endogenous reactivation or a combination of both. This persistence of Ab was also observed in 25 subjects at 20 years after a single vaccine dose in a clinical study conducted in Japan (92). All 25 subjects who were evaluated for humoral immunity via the fluorescent Ab to membrane Ag assay showed an Ab titer >8. However, in a much larger epidemiology study involving 350,000 subjects conducted by the CDC in collaboration with the Los Angeles County Department of Health Services (93), the varicella attack rates increased 36-fold from year 1 to year 9 after vaccination with just one dose, indicating substantial waning of immunity with time. The waning of Ab titer in adults within the first 10 years after vaccination was also clearly documented in one observational study that involved 461 healthy adults (94).

## Hepatitis A vaccine

HAVRIX manufactured by GlaxoSmithKline Biologicals is the first hepatitis A vaccine approved by the US FDA in 1995. Subsequently, VAQTA manufactured by Merck was approved by the FDA in 1996. Both formulations are sterile suspensions of purified hepatitis A virus inactivated with formalin and use aluminum-based adjuvants without preservatives. Based on the manufacturer’s product insert, HAVRIX contains aluminum hydroxide while VAQTA contains aluminum hydroxyphosphate sulfate. For both HAVRIX and VAQTA, two intramuscular doses of the vaccines have been recommended for children, adolescents and adults (95). Although the exact duration of protection after hepatitis A vaccination is unknown, the persistence of virus-specific Abs in vaccinees has been extensively studied in different age groups, even including a single-dose vaccination regime that was adopted by some countries (95). In fact, epidemiology studies conducted in Brazil revealed that even a single dose of VAQTA is highly effective, which reduced the incidence of hepatitis A in the entire country by 78% in three years (96). A clinical study conducted in Argentina involving 1088 children showed that 97.4% of all participants remained seropositive 6 to 9 years after a single dose of the vaccine (97). In a small-scale clinical study conducted by the CDC in Alaska that involved 183 participants for long-term follow-up (98), over 90% of children who were initially seronegative for hepatitis A remained seropositive at 10 years of age after two doses of HAVRIX vaccines administered during toddlerhood. However, it is worth noting that the virus-specific Ab titer dropped substantially within the first 7 years after vaccination and then decayed more slowly. In another long-term study conducted in Belgium that involved 187 participants, over 96% of subjects remained seropositive 17 years post two doses of HAVRIX (99). Mathematical modeling of the data suggested that the seropositive rate remained >95% for over 25 years, but a rapid decline in Ab titer during the first five years post vaccination was also clearly observed in this study (100). Further follow-up of this study showed that those subjects who had lost their seropositivity all mounted anamnestic responses after a booster shot (101), indicating the presence of long-lived virus-specific memory B cells.

## Human papillomavirus vaccine

Certain types of human papillomavirus (HPV) infections in humans can cause cancers in both men and women. GARDASIL manufactured by Merck is the first HPV vaccine in the US that was approved in 2006. This formulation contains the virus-like particles (VLPs) of the HPV produced from yeast *Saccharomyces cerevisiae*. The purified VLPs are then adsorbed onto aluminum hydroxyphosphate sulfate as the adjuvant for the vaccine. Vaccines prepared from different types of HPV VLPs can then be mixed to formulate multivalent vaccines to improve the protection coverage conferred by the vaccines. CERVARIX manufactured by GlaxoSmithKline Biologicals is the second HPV vaccine that was approved by the US FDA in 2009, although the company decided to cease the supply of this vaccine to the US market in 2016 due to very low demand. CERVARIX contains the HPV VLPs produced from Baculovirus instead of the yeast *Saccharomyces cerevisiae*. The purified VLPs are then adsorbed onto aluminum hydroxide. Furthermore, the adjuvant 3-O-desacyl-4’-monophosphoryl lipid A (MPLA) was also adsorbed onto aluminum hydroxide as an additional adjuvant in this formulation. For GARDASIL, each 0.5-ml dose contains roughly 20 to 40 μg of HPV type-specific L1 protein, the major capsid protein of HPV that self assembles into VLPs, together with 225 μg of alum adjuvant. In contrast, each 0.5-ml dose of CERVARIX contains 20 μg of HPV type-specific L1 protein together with 500 μg of alum and 50 μg of MPLA adjuvants. Because GARDASIL is already highly immunogenic and effective in the prevention of HPV-induced diseases (102), it left the question as to whether the extra alum and MPLA adjuvants in CERVARIX are necessary for a safe and effective vaccine. On the other hand, the reactogenicity associated with the use of MPLA has become a growing concern for the wider adoption of this vaccine by the general public (103). The persistence of protective immunity following immunization with HPV vaccines has been extensively studied in different regions around the world (104–108). Slifka and Amanna have also given an excellent and quantitative review on some of these studies (16). The consensus is that both 2-dose and 3-dose series can mediate long-term protection (over 10 years) and it is impressive to note that even a single dose of a bivalent CERVARIX vaccine can induce virus-specific Abs that are persistent for over 16 years post vaccination (109). Lastly, it is worth noting that the rapid but substantial decline of Ab titer during the first several years after vaccination has been reproducibly observed in different studies for both GARDASIL and CERVARIX (104, 105, 107, 108).

## Rotavirus vaccine

Rotavirus is the leading cause of severe acute diarrhea in children aged <5 years. There are two rotavirus vaccines available for infants in the US: RotaTeq manufactured by Merck with initial FDA approval in 2006 and ROTARIX manufactured by GlaxoSmithKline Biologicals with initial FDA approval in 2008 (110). Both RotaTeq and ROTARIX are live attenuated rotaviruses without additional adjuvants or preservatives. Specifically, RotaTeq is prepared from Vero cell cultures and contains five reassortant rotaviruses, while ROTARIX is derived from the human 89–12 strain of the rotavirus and prepared from Vero cell cultures. Both are administered orally, with three doses for RotaTeq and two doses for ROTARIX. Cases of rotavirus infection in the US have dropped significantly since the vaccine became available in 2006 (111). For RotaTeq, the effectiveness of this vaccine in the US has been estimated to be relatively stable between 76% and 89% over 5 years post vaccination (112), suggesting that waning of protective immunity is not substantial during this time frame. A similar trend in vaccine effectiveness over time was also reported for RotaTeq in Finland (113). However, a 12-79% reduction in vaccine efficacy 12 months after RotaTeq vaccination has been reported in Nicaragua (114). So, how could an identical vaccine perform so differently around the world? One important factor contributing to this phenomenon is the high incidence of rotavirus in resource-limited countries, which leads to natural immunity in the unvaccinated control group that biases the estimates of vaccine efficacy in vaccinees (115, 116). This phenomenon of apparent ‘waning’ immunity after RotaTeq vaccination in low-resource settings highlights the complications in epidemiology studies of vaccine efficacy, especially for vaccines that do not confer sterilizing immunity such as the current rotavirus vaccines.

## Zoster (Shingles) vaccine

The same virus that causes chickenpox in children can also cause shingles in adulthood upon virus reactivation, although it should be noted that the immune mechanisms needed for protection from shingles may differ from those for chickenpox. Specifically, the viral-specific cellular instead of humoral immunity has been found to be inversely correlated with the incidence of herpes zoster in clinical studies conducted in Japan (117). ZOSTAVAX manufactured by Merck was approved in the US in 2006 for the prevention of herpes zoster (shingles) in individuals 50 years of age or older. Not surprisingly, ZOSTAVAX is a live attenuated vaccine prepared using the same Oka/Merck strain of zoster virus that is used for making VARIVAX, the live attenuated vaccine indicated for varicella (chickenpox). This makes sense because both chickenpox and shingles are caused by the same virus. Both vaccines are produced from human diploid cell line MRC-5 and lyophilized with stabilizers without preservatives or other adjuvants. The major difference between ZOSTAVAX and VARIVAX is the dose of the live attenuated zoster virus. A 0.5-ml dose of reconstituted VARIVAX contains 1,350 plaque-forming units (PFU) of the live virus, while a 0.65-ml dose of reconstituted ZOSTAVAX contains 19,400 PFU of the same virus, which is almost 15-fold higher. As immunity wanes with age, this high dose of the vaccine might be necessary to stimulate protective immune responses in the elderly. Clinical trials on ZOSTAVAX showed that a single dose of this vaccine is effective in the reduction of herpes zoster and postherpetic neuralgia (a complication of zoster with persistent pain >90 days after the resolution of zoster rash) among the elderly (118). However, the overall efficacy of the vaccine declined rapidly one year post vaccination (119) and continued to decline substantially from 7 to 11 years post vaccination (120). In a separate observational study conducted in southern California, the effectiveness of ZOSTAVAX decreased from 68.7% in year 1 to 4.2% 7 years later (121). This trend in waning vaccine efficacy was also reported in another observational study conducted in northern California (122), although quantitative values of vaccine efficacy differed. Lastly, retrospective studies using data from Medicare have also uncovered this disturbing trend of waning protection after ZOSTAVAX vaccination (123). In summary, epidemiology data in the literature are quite strong in documenting the significant waning of immune protection within 10 years after ZOSTAVAX vaccination. This waning protection is particularly striking considering VARIVAX confers durable protection to pediatric populations against varicella (chickenpox) at a much lower concentration of the live virus, which has reduced the national incidence of varicella by 89% (52, 53). Besides the waning immunity of the elderly population, potential differences in mechanisms of protection may also be at play. Although this product was discontinued by Merck in 2020, if resumed, a booster dose of ZOSTAVAX should be seriously considered to mitigate the effects of waning protection after just one dose.

SHINGRIX manufactured by GlaxoSmithKline Biologicals is the second vaccine indicated for herpes zoster (shingles) approved by the US FDA in 2017 (124). Different from ZOSTAVAX, SHINGRIX is a subunit vaccine with novel adjuvants. The surface glycoprotein E (gE) of the zoster virus is overexpressed and purified from Chinese hamster ovary cells, which serves as the Ag in this vaccine. The adjuvant AS01_B_ is composed of 3-O-desacyl-4’-monophosphoryl lipid A (MPLA) from *Salmonella minnesota* and QS-21, a saponin purified from plant extract *Quillaja saponaria* Molina, which are combined in a liposomal formulation. Each 0.5-ml dose of SHINGRIX vaccine contains 50 µg gE, 50 µg MPLA, and 50 µg QS-21 without preservatives. This vaccine is recommended as two doses for intramuscular injection. It should be noted that the mechanisms of immune activation by SHINGRIX are likely to be very different from vaccines based on virion structures, because the gE Ag was supplied as a soluble protein instead of a particulate Ag. For comparison with ZOSTAVAX, we continue our discussion of SHINGRIX. In two large-scale clinical studies funded by GlaxoSmithKline Biologicals, SHINGRIX showed impressive efficacies of 97.2% overall for participants >50 years old (125), and 89.8% overall for participants >70 years old (126) in the reduction of herpes zoster and postherpetic neuralgia. A long-term follow-up study (127) showed that the efficacy of the two-dose SHINGRIX vaccine indeed dropped slightly with time. However, the vaccine remained 73.2% effective at 10 years after the initial two doses, which was correlated with the geometric mean concentration of anti-gE Ab in the sera. However, one should be open-minded that the mechanisms of protection could be cell-mediated instead of antibody-mediated. The apparent correlation with anti-gE Ab could be that Abs are a surrogate marker for an effective cell-mediated immune response. Moreover, one additional dose of SHINGRIX administered 10 years after the initial two doses elicited strong anamnestic Ab and CD4^+^ T cell responses (128), indicating that this vaccine remained highly effective. In addition to its immunogenicity and protective efficacy, the reactogenicity of the SHINGRIX vaccine is noteworthy. Beyond the common side effects associated with intramuscular injections, recipients of SHINGRIX have also reported significant systemic adverse reactions including fatigue (45%), headache (38%), shivering (27%), fever (21%) and gastrointestinal symptoms (17%). In contrast, none of these adverse reactions have been specifically associated with the administration of ZOSTAVAX. Therefore, these significant side effects from the SHINGRIX vaccine are likely associated with the use of the AS01_B_ adjuvants. These severe side effects may promote vaccine hesitance when time comes up for individual patients to decide what to do with zoster vaccines.

## Adenovirus vaccine

Adenoviruses in human populations are highly diverse and can cause a range of illnesses in people (129). Although vaccines indicated for adenovirus are not available to the general public in the US, the US FDA approved a live adenovirus vaccine in 2011 with exclusive use in military populations to prevent febrile acute respiratory disease caused by adenovirus types 4 and 7, because these viral infections can transmit easily in crowded settings such as military recruits (130). This vaccine contains live adenovirus types 4 and 7 prepared from human diploid fibroblast cell culture and is unattenuated. The viral preparations are lyophilized and formulated into enteric-coated tablets for oral immunization. In clinical trials, this vaccine was shown to be highly effective, with an efficacy of 99.3% and seroconversion rates above 93% for both adenovirus types 4 and 7 (131). A post marketing study for this vaccine also showed a very good safety profile when compared with a placebo group (132). A follow-up study showed that the vaccinees still maintained steady protective levels of neutralizing Abs specific for both types of vaccines 6 years post vaccination and no apparent decline in neutralizing Ab titers was observed (133). Therefore, this adenovirus vaccine can likely maintain durable protection for a long time with just one dose. As it turned out, this vaccine proved not only highly effective in clinical trials, but also reduced the adenovirus disease burden by 100-fold in a real world setting among military trainees, as demonstrated in a two-year observational study (134).

## Dengue vaccine

Dengue is a serious and growing public health problem in the world, especially in those endemic regions such as South America. DENGVAXIA manufactured by Sanofi Pasteur is the vaccine approved by the US FDA in 2019 for the prevention of dengue disease. DENGVAXIA is a tetravalent live chimeric virus vaccine for subcutaneous injection. The chimeric virus was constructed using the yellow fever virus strain 17D-204 as the backbone, in which the genes for yellow fever virus envelope proteins were replaced with those of dengue viruses. The formulation does not contain preservatives or other adjuvants. However, it is only approved for use in individuals 9 through 16 years of age with lab-confirmed prior dengue infection and living in endemic regions (135). The reason is that DENGVAXIA vaccination will increase the risk of hospitalization and severe dengue in those naïve individuals upon their first natural dengue virus infection (136). This may sound very strange at first: how could a vaccine exacerbate disease outcomes instead of conferring protection? There have been extensive studies into this phenomenon in literature and the mechanisms remain under active investigation. The prevalent model to explain this phenomenon is Ab-dependent enhancement (137–139), although T cell-mediated immunopathology was also suspected (140). Ab-dependent enhancement of viral infectivity is not a new phenomenon in virology. Instead, it has been well documented in literature for more than 60 years (141). At molecular and cellular level, this phenomenon occurs when neutralizing Abs are at suboptimal concentrations and not able to completely neutralize the infectivity of viruses (142). The IgG molecules bound on virion surfaces can bind to Fc receptors on cell surfaces to enhance virion attachment (143). If these cells are permissible for viral entry and replication, this enhanced attachment will lead to elevated infection by the incompletely neutralized viruses. In reality this phenomenon may well occur under a specific range of IgG concentrations, because a threshold number of virus-cell attachment points is typically required for optimal infection (144). This may also help explain why a very low titer of IgG does not promote severe dengue disease, an outcome which has been observed in epidemiology studies (138). DENGVAXIA offers a great example which illustrates the significance of Ab concentration in vaccine-mediated protection. This concentration needs to be maintained at sufficiently high levels to effectively neutralize infectious virions completely. Otherwise, the vaccine may exacerbate the disease instead of protecting against it. Lastly, based on a recent study, the protective effect of DENGVAXIA was mainly observed in the first three years post vaccination (145), suggesting a waning of Ab with time below the level needed for protection. This is consistent with an earlier study showing low dengue Ab titers 5 years after vaccination (146).

## Chikungunya vaccine

The US FDA approved two chikungunya vaccines recently for the prevention of disease caused by the chikungunya virus, a mosquito-borne alphavirus that is endemic in many parts of the world (147). IXCHIQ manufactured by Valneva Scotland is a live attenuated virus produced from Vero cells, purified and lyophilized for intramuscular injections upon reconstitution. This vaccine does not contain preservatives or other engineered adjuvants. A single dose of the vaccine is recommended for individuals 18 years of age or older. In two separate trials of live attenuated chikungunya vaccines, 85 and 100% of vaccinees, respectively, remained seropositive one year after receiving a single injection of the vaccines (148, 149). VIMKUNYA manufactured by Bavarian Nordic A/S consists of recombinant chikungunya VLPs (150) produced from HEK293 cells, purified and adsorbed onto aluminum hydroxide as adjuvant for intramuscular injections. A single dose of VIMKUNYA is recommended for individuals 12 years of age or older. In a phase 2 trial of the VLP vaccine, even two doses of unadjuvanted VLPs without alum elicited viral-specific neutralization titers that were comparable to adjuvanted groups, and the sera neutralizing Abs were durable up to 2 years of the monitoring period (151).

## Lessons learned from the durability of antiviral vaccines

### Being ‘live’ is not always better

The 18 antiviral vaccines that were approved by the US FDA over the last century as we have reviewed above provide ample examples of varying degrees of vaccine efficacy and durability from different vaccine platforms. Space limitation does not allow us to dive into the diverse biology and detailed structures of these viruses (152) that are important for the outcome of a vaccine. However, there are several important implications from this list of vaccines that we shall discuss. First, while we agree that there are many excellent examples of live vaccines that confer robust and durable protection, live vaccines do not always perform better than inactivated or subunit vaccines, as exemplified by FluMist in adult populations where there were head-to-head comparisons available. There are more examples. The varicella-zoster virus causes shingles in the elderly upon viral reactivation. A single dose of the live attenuated vaccine ZOSTAVAX has poor durability of protection against shingles in the elderly. In contrast, the subunit vaccine SHINGRIX offers excellent long-term protection against shingles. Therefore, it is a misconception that live attenuated vaccines always perform better than inactivated or subunit vaccines in efficacy and durability. Moreover, DENGVAXIA is a live chimeric virus vaccine. This vaccine may not fall directly into the category of ‘attenuated’ vaccines, because the construction of this vaccine involved a tropism change in the original virus. Nevertheless, this live vaccine protects against dengue with poor durability. Importantly, the subthreshold concentration of anti-dengue IgG elicited by this live vaccine can further exacerbate the disease upon the vaccinee’s first natural exposure to dengue virus. Lastly, live attenuated vaccines can expose the public to a low but definitive risk of severe adverse effects due to viral replication or virulence reversal, exemplified by the yellow fever vaccine and the oral polio vaccine.

### Mechanisms of inactivated virus vaccines without exogenous adjuvants

The second important lesson that we have learned is that to be an effective vaccine that elicits potent and long-lasting protection, it is not necessary to have live viruses. Inactivated viruses can do an excellent job. In this regard, it is noteworthy to pay attention to the inactivated vaccines that have conferred effective protection for at least two years, especially those vaccines that do not have any apparent exogenous or engineered adjuvants in their formulations. These include the whole inactivated influenza vaccine (not the split virus), the inactivated polio vaccine, the inactivated rabies vaccine, and the inactivated Japanese encephalitis vaccine JE-VAX (**Figure 1**).

The absence of exogenous or engineered adjuvants in these four vaccines makes it possible to rationalize the mechanisms of protection based on the immunogenicity of the inactivated virions, which is not possible when exogenous adjuvants are added into their formulations. For inactivated virion particles, while there are reported Ag-specific CD8^+^ T cell responses (153, 154), the lack of viral replication in host cells often hinders the induction of Ag-specific CD8^+^ T cells (155–157). However, virion particles alone, in the absence of any viral replication, are potent inducers of B cell Ab responses (17, 158), the mechanisms of which have been studied in depth especially recently in mice (159–163).

**Figure 2:**
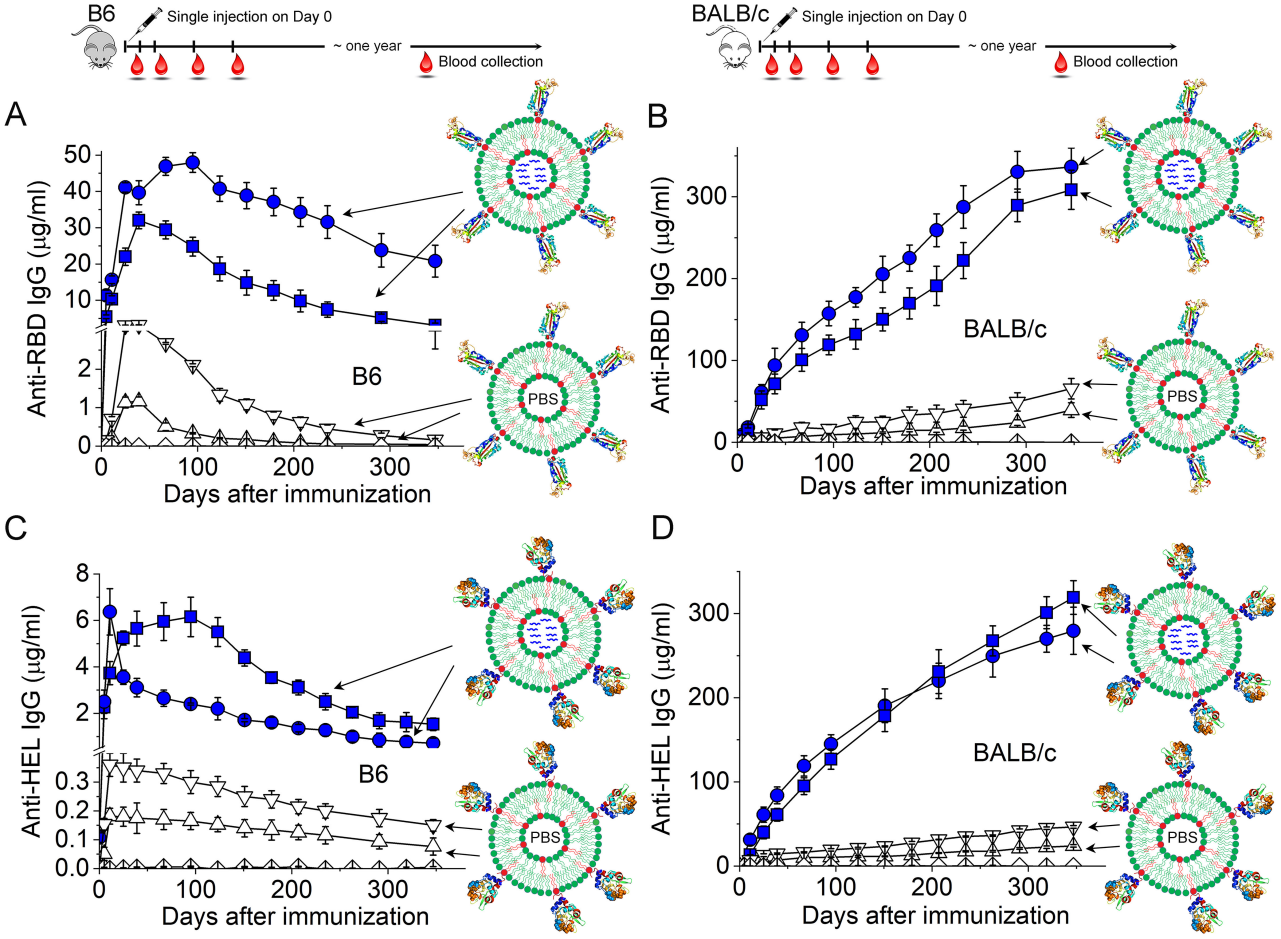
Duration of the IgG responses in C57BL/6 (B6) or BALB/c mice induced by a single injection of SVLS at sub-microgram doses without additional adjuvants. **(A–D)** Concentrations of Ag-specific IgG in mouse sera collected on different days after a single injection of various SVLS agents, where **(A, B)** are for anti-RBD IgG in B6 **(A)** and BALB/c mice **(B)** upon immunization with SARS-CoV-2 RBD conjugated SVLS, respectively, and **(C, D)** are for anti-HEL IgG in B6 **(C)** and BALB/c mice **(D)** upon immunization with hen egg lysozyme (HEL) conjugated SVLS, respectively. As schematically shown in each panel, SVLS are liposome-based structures of 120-nm diameters with site-specific conjugation of protein Ags on the surface and optional encapsulation of nucleic acids or T-cell epitope peptides within these structures. We define the average number of protein Ag molecules per structure as the epitope density (ED). Specifically, blue squares and blue circles in **(A, B)** were from RBD-conjugated SVLS of varied ED and encapsulating a 20-mer single-stranded DNA containing two CpG dinucleotide motifs (DNA1), as represented by the blue wavy lines within these structures. Upper and downward triangles in **(A, B)** were from RBD-conjugated SVLS of varied ED with phosphate buffered saline (PBS) within these structures. Blue squares and blue circles in **(C, D)** were from HEL-conjugated SVLS of varied ED and encapsulating DNA1 within these structures. Upper and downward triangles in **(C, D)** were from HEL-conjugated SVLS of varied ED with PBS within these structures. Diamonds throughout all panels represent data from mice immunized with control SVLS. All concentrations were measured using ELISA based on standard curves obtained from reference monoclonal Abs. Throughout this figure, N=4 for each time point [Adapted from (162)].

Two features of virion particles are responsible for this Ab response. First, the ordered display of surface Ag on virions can serve as a stand-alone danger signal, akin to all viruses, to activate Ag-specific germline B cells for proliferation, differentiation, class-switch recombination and seeding of long-term IgG response, although the concentration of antiviral IgG induced by this multivalent Ag display is low (161–163). The detailed biophysical features of these four virions that convey the stand-alone danger signals have been reviewed previously (164). Second, upon internalization of virion particles mediated by B cell Ag receptors (BCRs), the nucleic acid genomes inside virions can further activate endosomal Toll-like receptors (TLRs) to dramatically influence B cell differentiation, germinal center responses (159) and the magnitude of IgG in both the short (163) and long term (162), which leads to high concentrations of potent antiviral IgG. As shown in **Figure 2**, by taking advantage of synthetic virus-like structures (SVLS) that we recently developed using highly purified biochemical ingredients (165, 166), we showed that protein Ags alone arrayed on the surface of a virion-sized scaffold are sufficient to seed a low-amplitude but long-lasting Ab response in the absence of any other adjuvants (open triangles in **Figures 2A–D**) (162). The presence of nucleic acids internal to these structures, on the other hand, can dramatically modulate the magnitude of the long-term Ab response, as shown by the inclusion of a CpG-containing DNA within these structures (blue symbols in **Figures 2A–D**). It is noteworthy that a single injection of SVLS at submicrogram Ag doses without exogenous adjuvants is sufficient to produce these long-lasting IgGs in mice.

**Figure 3:**
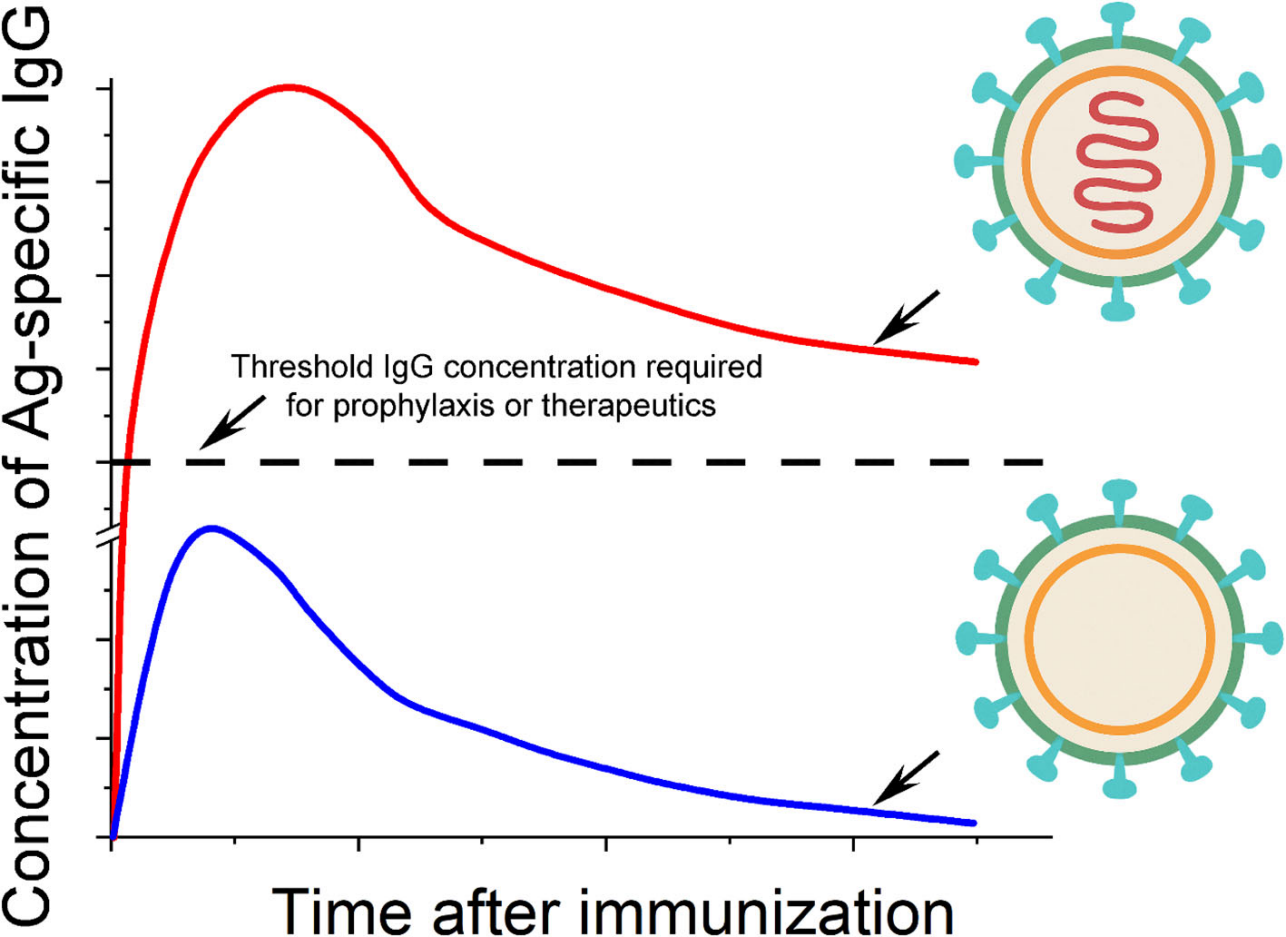
Molecular models of viral immunogenicity and the kinetics of IgG induced by these structures. There are two essential elements to the immunogenicity of a typical virion in general: the oriented display of viral surface protein antigens (shown in cyan) on a virus-sized scaffold (green and yellow shells), and internal nucleic acids (shown in red) with native phosphodiester backbones that reside in the interior of the scaffold structure. The ‘shell’ of the virion as shown in the lower portion of the figure typically elicits Ag-specific IgG that is below the threshold of IgG required for either prophylaxis or therapeutics. The inclusion of internal nucleic acids can substantially increase the concentration of IgG to be well above the level of IgG needed for protection or therapeutics.

These studies have important implications for understanding the immunogenicity of viruses in general. In the context of these four different virions that we have emphasized above (influenza virus, polio virus, rabies virus and the Japanese encephalitis virus), we hypothesize that surface Ag display on these virions, alone, seeds a long-term Ab response, and that TLR activation by the nucleic acid genomes inside these virions amplifies the magnitudes of the Ab response in both the short and long term. Supporting this hypothesis, a recent clinical study conducted in Hong Kong revealed an apparent association between the titer of anti-hemagglutinin post vaccination and the single nucleotide polymorphisms of TLR7 and TLR8 among 550 children participants (167). It should be noted that even though a ‘split virus’ vaccine was followed in this study, viral genomic RNA likely remained in the vaccine that can explain the gene association studies due to formaldehyde inactivation that preceded virion disruption, in which RNA and protein would be covalently crosslinked. The detailed mechanistic pictures that resulted in the long-term protection by these four vaccines may differ slightly from one another, because these virions have very different surface protein Ags and genomes of different sizes and sequences. For example, the magnitude of CD4^+^ T cell help, which is specific to each protein Ag, and the extent of TLR activation, which is specific to each nucleic acid type and sequence, could differ from one another. However, the consistent observation of the long-lasting antiviral IgGs from these four vaccines in human populations and the mouse studies above argue strongly for a mechanistic common ground from mice to humans: a virus-like structure — absent of exogenous adjuvants—is all that is needed for a long-lasting IgG response (**Figure 3**).

This virus-like structure comprises two essential features: (1) the oriented display of viral surface protein Ags on a virus-sized scaffold, and (2) internal nucleic acids (iNAs) with native phosphodiester backbones. In detail, the orientation of the protein Ag is defined with regard to its N- or C-terminus and its attachment or anchoring point on the scaffold. For the SVLS platform, a site-specific engineered Cys close to the C-terminus of the protein Ag mediates all the covalent attachment of the protein on the surface of a liposome (165, 166). This is to mimic viral protein Ags on the surface of virions that are always orientation specific. This orientation specificity is a key feature for B cell Ab response because it allows quantitative definition of the spatial density of surface epitopes (168). The variation of the epitope density is known to influence the B cell Ab response both qualitatively and quantitatively (17, 169, 170). In our model, a high epitope density is not required for potent Ab response in mice. Provided that a potent TLR ligand is encapsulated within the virus-like structure, as low as 5 epitopes per particle can induce potent Ag-specific IgG in mice due to compensatory signaling between BCR and TLR (163). The virion-sized scaffold can be as simple as a nonimmunogenic lipid bilayer vesicle, as we showed in mice for SVLS with a diameter of ~120 nm (163). This scaffold for the poliovirus is a protein cage of 31 nm in diameter that is made of viral VP1, VP2 and VP3 proteins (171), which is the smallest among the four inactivated human virus vaccines. The iNAs are nucleic acids that reside in the interior of the lipid vesicle or a protein cage, which can be either DNA or RNA with native phosphodiester backbones, such as genomes in virions. In SVLS, these are short DNA or RNA oligos for the convenience of encapsulation within lipid vesicles. Importantly, the iNAs are protected by the scaffold from nuclease degradation (165, 166) until these structures are internalized by the Ag-specific B cells. Neither do iNAs leak readily from the scaffold structures, which mark an important distinction from porous polymer scaffolds such as poly(lactic-co-glycolic acid) (172). The ready leakage of nucleic acids from the scaffold structures may lead to non-specific inflammation *in vivo* (173) and evoke different mechanisms of immune system activation.

A virus-sized scaffold featuring multivalent display of protein Ags on its surface is immunogenic on its own (**Figure 3** bottom inset). The mechanisms by which this structure activates Ag-specific B cells without iNAs were revealed by our recent studies using SVLS. Specifically, BCR activation triggered by SVLS can bypass the LYN-dependent inhibitory tone and elicit robust and prolonged Ca^2+^ signaling (161). Notably, these activated B cells can undergo robust NF-κB activation that is completely independent of MyD88 or IRAK1/4 (161). *In vivo*, this type of structure alone in the absence of iNAs can trigger Ag-specific B cells for proliferation, differentiation, class-switch recombination, secretion of IgG and also seeding of long-term IgG response (162, 163). However, the titer of the resulting IgG is poor, i.e., it only triggers limited IgG secretion, resulting in low concentrations of IgG. This is supported by the studies in mice using SVLS (162, 163) and studies in rhesus macaques using computationally designed self-assembling protein nanomaterials (174), the latter of which present Ags on a more rigid oligomeric protein scaffold than the liposome-based SVLS. The presence of iNAs (**Figure 3** top inset) substantially elevates the immunogenicity of the structure and elicits potent and durable Ag-specific IgG at high concentrations. In our data collected from mice, this difference in Ab concentrations with or without iNAs can easily be over 100-fold in the long term (162). Moreover, this robust IgG induction occurs at a much lower Ag dose and a much lower quantity of nucleic acids compared to other forms of immunogens in literature (162, 172). The B-cell intrinsic TLR activation allows us to use only 0.1 to 1.0 µg of CpG DNA per mouse for immunization (162, 163), which is more than 20-fold lower than the dose of CpG DNA typically used in mouse studies (175, 176). Importantly, only nucleic acids with native phosphodiester backbones are needed, as stability-enhancing modifications to nucleic acids have been linked to off-target intracellular interactions *in vitro* (177, 178) and toxicity *in vivo* (179).

It should be noted that the impact of iNAs will not manifest unless they are located inside the virus-like structures, consistent with B cell intrinsic TLR activation. Control experiments that we did revealed that the self-adjuvanting effects of iNA were completely lost if they were removed from the interior of these structures and delivered instead as an admixture of external nucleic acids and empty SVLS without iNAs (163). This result also emphasizes that mechanistically it is critical for the Ag-specific germline B cell to receive both BCR and TLR stimulation signals at a single cell level, i.e., a B cell is activated by the multivalent display of protein Ags on the surface of virions, and the same B cell needs to receive TLR activation signals following BCR-mediated endocytosis to undergo further activation. Moreover, these two signals of activation integrate within the single Ag-specific B cell, as evidenced by the compensatory signaling between the BCR and TLR in the elicitation of Ag-specific IgG by SVLS (163).

We should emphasize that the clarity of the immunogen composition is critical for our analysis here. The lack of exogenous adjuvants in these four human vaccines allowed us to rationalize their long-term efficacies based on the immunogenicity of these inactivated virions, which is not possible if exogenous adjuvants were included in these vaccines. This is because the effects of adjuvants on immune responses are often multifaceted or under characterized (180). Therefore, the mechanisms of B cell activation and resulting Ab responses are likely to be very different compared to scenarios without those added exogenous adjuvants. As a result, it would be difficult to interpret the long-term Ab responses using a common set of mechanisms.

Further support for this model of viral immunogenicity in relation to the long-term IgG response comes from investigation of vaccines listed in **Table 1** that are based on supramolecular structures of virions but incorporated exogenous adjuvants in their final formulations. These are hepatitis B subvirion particle vaccines, hepatitis A inactivated virus vaccines, and HPV vaccines based on virus-like particles (VLPs). Among these three, hepatitis B virus subvirion particles are self-assembled from HBsAg protein. Recent high-resolution structural analyses of these particles have not revealed the presence of any nucleic acids but lipid moieties in these structures (181, 182). Based on our model (**Figure 3**), even though these structures themselves present highly repetitive epitopes on a virus-sized scaffold, these structures by themselves are not sufficient to elicit high concentrations of antiviral IgG. In fact, this was shown to be the case in a recent comparative immunization study in rhesus macaques (183): after three doses of intramuscular immunizations of an adjuvant-free HBsAg vaccine, only one out of four rhesus macaques developed anti-HBs Ab above the threshold level of protection (10 mIU/ml). Therefore, it makes sense to include additional adjuvants in this vaccine (alum in this case) to enhance the immunogenicity of these structures for robust and durable protection.

The HPV vaccines are VLPs adjuvanted with alum. These HPV VLPs are self-assembled from the major capsid protein L1 and devoid of nucleic acids based on a high-resolution crystal structure reported for one of these VLPs (184). A study from Merck showed that unadjuvanted HPV VLPs induced ~tenfold lower HPV-specific IgG titer intradermally than the vaccine adjuvated with alum at the same dose through intramuscular injection in rhesus macaques (185). In contrast, substantial dose sparing by intradermal delivery in clinical settings has been well documented for inactivated influenza vaccines (186), inactivated rabies vaccines (187), and the live-attenuated ZOSTAVAX vaccine (188). Although a head-to-head comparison between HPV VLPs with or without alum administered via the same route is necessary to draw conclusions, these data suggest that HPV VLPs are likely to be weakly immunogenic on their own and consistent with our model presented in **Figure 3**.

Marketed hepatitis A vaccine is an inactivated virus adjuvanted with alum. Hepatitis A virus belongs to the same family of picornaviridae as the polio virus. Infectious hepatitis A virions harbor a positive sense single-stranded RNA (ssRNA) genome of ~7.5 kb (189). Based on our model of viral immunogenicity in relation to the long-term antiviral IgG above (**Figure 3**), inactivated hepatitis A viruses by themselves should be able to elicit robust and durable antiviral IgG, because the icosahedral virion surface presents repeated epitopes at high spatial density and the ssRNA genome inside the virion can activate endosomal TLR7 upon internalization by the virus-specific B cells. So, is it necessary to use Alum as an adjuvant for this vaccine? Interestingly, in an early preclinical study reported in 1986, new world owl monkeys (*Aotus trivirgatus*) withstood the challenge of infectious hepatitis A virions after vaccination with inactivated viruses without additional adjuvants (190), demonstrating that an inactivated hepatitis A vaccine was sufficient to elicit protective immunity without alum. In a separate preclinical study conducted using guinea-pigs, the investigators compared Ab responses among animals immunized with a plain inactivated hepatitis A vaccine, or the same inactivated vaccine adjuvanted with 0.5 mg Al(OH)_3_, 1 mg Al(OH)_3_, or 0.3 mg AlPO_4_ respectively (191). As it turned out, the mean Ab titers were comparable among all experimental groups, although AlPO_4_ afforded higher rates of seroconversion at low doses of Ag. In other words, the addition of alum to inactivated hepatitis A vaccine did not substantially boost the titer of antiviral IgG, indicating that a plain inactivated hepatitis A vaccine is sufficient to elicit highly protective Ab responses without additional adjuvants.

Lastly, we would also like to comment on the newly approved chikungunya VLP vaccine considering the recent interesting clinical data. In this phase 2 randomized, double-blind clinical trial, two doses of unadjuvanted VLPs without alum elicited viral-specific neutralization titers that were comparable to VLPs adjuvanted with 300 µg aluminum hydroxide; and even a single dose of 40 µg adjuvanted VLPs elicited comparable neutralization titers that were durable up to 2 years of the study (151). In the literature, this VLP vaccine has been cited as particles without genetic materials (192). Is this VLP truly highly immunogenic on its own without any nucleic acids that apparently violates our model of immunogenicity presented in **Figure 3**? For this we have investigated the structures of these VLPs obtained from cryo-electron microscopy maps by two different labs (193, 194). In both structural studies, electron dense cores in these VLP structures have been clearly identified and designated as RNAs. These VLPs are known to package cellular RNAs in their cores in place of the viral genomic RNA when they are produced from expression cell lines (195). *In vitro* studies using purified alphavirus nucleocapsid proteins have demonstrated the requirement of nucleic acids to initiate the virion core assembly (196, 197). Therefore, we think that these chikungunya VLP vaccines produced from HEK293 cells in fact contain cellular RNAs in their cores, which serve as potent adjuvants for these vaccines. Future studies to quantify the amount of RNA in these vaccines will help address this question. In summary, all the evidence in the vaccine literature that we have reviewed above fully supports our model of viral immunogenicity for robust and durable antiviral IgGs (**Figure 3**).

### RNA or DNA? - the natural adjuvants for long-term Ab responses

In our model shown in **Figure 3**, it is critical to have TLR activation downstream of BCR activation within the same Ag-specific B cells. This dual activation will lead to Ab affinity maturation and also durable IgG as we showed recently in mice (162). The B cell intrinsic TLR7 activation is also required for neutralizing Ab responses to SARS-CoV-2 in mice (198). There are multiple nucleic acid sensing TLRs that are important for both antiviral responses and autoimmunity (199–202). Besides ligand differences, are there quantitative differences among them for the enhancement of Ab responses? At this point, we don’t have answers to this question and in fact we understand very little in this regard. However, it has not escaped our attention that all four inactivated human vaccines as we discussed above contain ssRNA as their viral genomes. Specifically, the influenza virus encapsulates 8 negative sense ssRNA segments which total ~ 14 kb inside the virion (203). The polio virus harbors a positive sense ssRNA genome of ~ 7.4 kb (204). The rabies virus carries an unsegmented negative sense ssRNA genome of ~ 12kb in length (205), while the Japanese encephalitis virus contains a positive sense ssRNA genome of ~ 11 kb (206). Lastly as mentioned above, a plain inactivated hepatitis A vaccine can mediate protection in nonhuman primates without additional adjuvants (190), in which the hepatitis A virion contains a positive sense ssRNA genome of ~ 7.5 kb (189). Is it coincidental that they all happen to be RNA viruses, or does TLR7 activation by ssRNA trigger a robust IgG response in humans? The answer to this question can be potentially addressed using our SVLS platform and experimenting with nonhuman primates. However, for a given animal species, the answer to this question is likely shaped by evolutionary exposure of both viral and self-antigens in order to achieve a fine balance between robust antiviral responses and attenuation of undesired autoantibody responses. Because 62% of all human viruses identified to date use RNA as viral genetic materials (207), the preponderance of these effective inactivated RNA viral vaccines may be naturally biased by the human virome. The situation could be very different in mice. For example, by sequencing of viral DNA or RNA in the feces collected from wild rodents in the US, 91% of these viruses were identified to be single-stranded DNA viruses (208). Despite being homologous proteins both expressed in B cells, studies in mice have showed that TLR7 and TLR9 manifest distinct regulation (209, 210) and have opposing roles (211–214) in mouse models of autoimmune disease, with TLR7 promoting (215) while TLR9 negatively regulating (216, 217) disease pathogenesis. Given their distinct regulation and different roles in autoimmunity, their functions in antiviral responses are warranted for further dissection and investigation. Different species have exposures to different viromes during evolution. Different species also have different expression patterns for TLRs (218, 219). Thus, results from one species should always be interpreted with caution for their relevance to the other. Lastly, the search within these viral RNA genomes for potential sequences of high potency in the activation of human TLR7 is also warranted.

### The kinetics of the IgG response induced by antiviral vaccines

The kinetics of Ab responses as we have reviewed above for human antiviral vaccines are similar to those we observed in C57BL/6 mice upon immunization with SVLS (162). This is schematically shown as the red curve in **Figure 3**, in which there was a rapid burst of antiviral IgG within the first few weeks of immunization, followed by a peak IgG concentration, and then decay with time. In both mice and humans, these kinetics of antiviral IgG can be achieved with a single dose of an immunogen, for example SVLS immunization in C57BL/6 mice (162) and CERVARIX vaccination among women from Costa Rica (106), although in many cases of human vaccines, multiple doses of the vaccine were needed. For human vaccines, these decay kinetics have been been quantitatively analyzed in order to obtain information on potential mechanisms of decay (16). Based on what we have reviewed above, this phenomenon of antiviral IgG decay with time appears unavoidable in humans, no matter how fast or how slow it decays, which is true even for some highly effective live vaccines such as smallpox vaccines, yellow fever vaccines, MMR and chickenpox vaccines. However, it is encouraging that the kinetics of IgG decay are usually composed of multiple phases, with an initial fast decay followed by a much slower decay with time. For example, as we have described above for several vaccines, initial IgG decay was very fast within the first several years of completing the vaccine series. These fast decays suggest that the primary vaccine series produced many plasma cells that were not very long-lived, which vanished quickly with time. For a vaccine to be effective and durable, it is essential that during the later slow phase of IgG decay, the concentration of anti-viral IgG remains above the threshold concentration needed for protection, as indicated by the black dashed line in **Figure 3**. The physical concentration of an Ab after immunization is highly significant for a prophylactic antiviral vaccine. It is well demonstrated that above a minimum avidity threshold, protection *in vivo* simply depended on a minimum concentration of the Ab in the serum (220). As we have reviewed above, the right level of Ab concentration is even more critical when antibody-dependent enhancement occurs, as demonstrated by Katzelnick et al. for DENGVAXIA (138). In our recent comparison between SVLS and Qβ bacteriophage virus-like particles (VLPs), we found that SVLS encapsulating a CpG-containing DNA can elicit a higher concentration of Ag-specific IgG than Qβ VLPs in mice (221). The cellular mechanisms behind this difference remain to be investigated. However, this result suggests that the SVLS platform has the potential to be further explored, especially to achieve a specific concentration for the IgG of interest for the long-term. The data we obtained using SVLS in mice suggest that by adjusting TLR activation we can modulate this concentration of Ab (162, 163). The ability to tune this final plateau of IgG concentration is certainly highly desired for both prophylactic and therapeutic development that relies on Ag-specific Abs.

## Cellular mechanisms that give rise to long-lasting IgG

The cellular mechanisms that give rise to long-lasting IgG have been extensively studied in the past two decades (222–224), which are not the focus of the current work. However, the clarification of the molecular compositions of an immunogen that can elicit long-lasting IgG in both mice and humans has important implications regarding the cellular mechanisms for durable IgG induction. The presence of long-lasting IgG in mice after immunization using SVLS without any iNAs (162) suggests that the multivalent Ag scaffold alone, in the absence of any other adjuvants or TLR activation, might seed the production of long-lived plasma cells (LLPCs), although the low concentration of the Ag-specific IgG indicates that the number of LLPCs is low. This result is fully consistent with the imprinted lifespan model proposed by Amanna and Slifka, in which plasma cells are “imprinted with a predetermined lifespan based on the magnitude of B cell signaling that occurs during the induction of the Ab response” (225). Our recent work using SVLS to examine the signal transduction events upon B cell encounter of Ags of different biophysical forms revealed that there are qualitative differences between SVLS and a soluble Ag counterpart early on upon BCR activation (161), which also supports this model. As we have mentioned above, a virus-like particulate Ag such as SVLS can efficiently evade the inhibitory signaling mediated by the LYN kinase, trigger durable Ca^2+^ signaling and robust NF-κB activation for extensive B cell proliferation, all in the absence of iNAs or cognate CD4^+^ T cell help. It remains to be determined in the future if these qualitive differences early on from BCR activation may eventually translate into the ‘lifespan’ of the resulting plasma cells. But overall, these data support the model that the formation of LLPCs was ‘imprinted’ by the multivalent display of epitopes on a virus-sized scaffold. Among others, the role of iNAs and intrinsic TLR activation is likely to substantially increase the number of these LLPCs or the capacity of LLPCs to secrete IgG, which can be tested. While it is almost certain that long-lasting IgG requires the deposition of plasma cells in the bone marrow niche, the mere presence within the bone marrow does not guarantee that these plasma cells will all be long-lived due to the heterogeneity of these cell populations (15, 226, 227). It will be interesting in the future to characterize the plasma cells induced by SVLS in mice, and plasma cells induced by the above four human vaccines in **Figure 1** to determine if there are any common markers that can be used to identify these important cell populations for long-term humoral immunity (228, 229).

Slifka and Amanna have also proposed the role of antigenic threshold in the induction of protective Ab responses (16). Our model for viral immunogenicity (**Figure 3**) is fully consistent with their model. Moreover, the literature on inactivated human vaccines in conjunction with our own recent work allows us to specify what is the ‘antigenic threshold’. In our view, at minimum, this ‘antigenic threshold’ has three parts: (1) the physical number of viral or virus-like particulate Ags, which contributes to the dose of the Ag, (2) the potency of the multivalent display of proteins on virus-sized scaffold; and (3) the costimulatory signal to Ag-specific B cells provided by iNAs that reside within the particulate Ags. Co-stimulation of the Ag-specific B cells by both the multivalent Ag and iNAs is the key to a robust and durable IgG response. Based on the current knowledge, the potency of the multivalent Ag display on a virus-sized scaffold will be contributed by the average spatial density of the epitope on the scaffold (163, 230), the affinity of the BCR towards the epitope, the frequency of the Ag-specific precursor B cells (231) and the ability of the Ag to recruit linked CD4^+^ T cell help. Our specification of the ‘antigenic threshold’ is also applicable to live or attenuated vaccines, which can undergo full or limited viral replication and consequently, the physical number of particulate Ags can increase with time after immunization, which increases Ag dose before they are eventually neutralized.

Lastly, while we have focused on the common features in plasma antibody responses between mice and humans above, it is worth noting one important difference between these two species, i.e., the distinct average lifespans. Inbred lab mice have an average lifespan of ~ 2 years (232), which is much shorter than the global life expectancy of 71.4 years (https://data.who.int/indicators/i/90E2E48 updated on Aug 2, 2024). Therefore, evolutional pressures are not in place for mice to evolve long-lived plasma cells that rival human lifespan. As a result, one should execute caution when considering common cellular mechanisms for long-lasting IgG between these two species. While our model in **Figure 3** applies to both species, we have not made a clear distinction between short-lived versus long-lived plasma cells in their contributions to this model shown in **Figure 3**.

## Comparison among different vaccine platforms

Live attenuated viruses can serve as outstanding vaccines in many cases, exemplified by the MMR vaccine. However, to make a live attenuated vaccine with robust and durable protection is likely to be highly empirical, because on the one hand the vaccine needs to elicit potent and long-lasting IgG, and on the other hand, the vaccine needs to be less virulent and has no ability to revert to the virulent strains. This is critical for the vaccine safety and may have to involve highly empirical virus-specific changes or manipulations. From the current literature review, it is clear that a live vaccine is not a necessity for effective and durable protection, as evidenced by the inactivated viral vaccines that have been marketed successfully and globally for various viral diseases.

Moreover, we would also like to emphasize an important difference between live vaccines and inactivated vaccines that should be taken into account for their efficacy comparison. Many people are aware of the hallmark difference between these two vaccine platforms, which lies in their biological capabilities to replicate within the host species. However, in many cases people have overlooked one major difference between them, which is epitope modification. A chemistry-based critical thinking is needed here. Whereas live vaccines always carry epitopes in their native conformations, inactivated viruses always carry modified epitopes. The inactivation process using formaldehyde is a two-step chemical reaction. First, formaldehyde reacts with amino groups (especially the ϵ-amino group of lysine) to form a hydroxymethyl adduct. Second, the hydroxymethyl group can then react with another amino group to form a methylene ‘bridge’. These changes can dramatically influence recognition of viral surface Ags by B cells. For example, it has been shown for inactivated polio virus vaccines that epitopes on type 2 and 3 polio viruses in this vaccine were modified by the inactivation process to such an extent that some monoclonal Abs that recognize the infectious strain failed to bind to the inactivated virus in the vaccine (233). Therefore, this process is highly undesired for targeting specific Ags. Given this context of epitope modification, it is remarkable to note that there are several inactivated vaccines that are highly effective and durable in their protection in the human population, as we have discussed in this manuscript. Along this line of vaccine development, new techniques such as hydrogen peroxide-based virion inactivation have been shown to better preserve antigenic structures while efficiently inactivating virion infectivity (153), the continued development of which has provided highly encouraging preclinical data for several inactivated vaccines (234–236).

Compared to inactivated vaccine platforms, the platform of SVLS could bring advantages to the field of vaccinology if successful. First, there is no need to inactivate any ‘infectivity’ because the structure itself is noninfectious and thus no need to introduce agents such as formaldehyde or β-propiolactone, both of which are actually carcinogens. Specifically, SVLS use recombinant proteins purified in their native conformations and preserve important antigenic structures, which would be critical for neutralization, and allows researchers to fully explore the contemporary technology of recombinant protein engineering for optimization of a specific immunogen. In contrast, either formaldehyde or β-propiolactone inactivation of viruses will inadvertently modify viral surface proteins or even destroy surface epitopes.

Second, SVLS use the type of molecules that already exist in the human body as the ‘natural’ adjuvants and allow us to explore them for potent Ab responses. This is in sharp contrast to alum and other recent adjuvants such as MPLA. Aluminum is non-essential to the human body and potentially toxic especially at elevated levels. Because the human body does not need aluminum, the alum adjuvants have to be removed by several elimination mechanisms in the body. There have been rigorous studies on the safety and elimination of aluminum adjuvants in animal models and infants (237–239). With a better understanding of viral immunogenicity, the potential utility of different DNA or RNA sequences as ‘natural’ adjuvants remains to be explored in the future, which has its solid footing on fundamental mechanisms that mother nature has evolved to cope with viral infections and attenuate autoimmunity.

Lastly, the SVLS platform displays Ags in a lipid bilayer environment where the Ags can undergo two-dimensional lateral diffusion. Although rigid Ag display is not essential for the immunogenicity of these structures in mice from our recent comparative studies (221), how important the rigidity of Ag attachment is for immunogenicity in humans remains to be determined. Among the four inactivated human vaccines that have been marketed without exogenous or engineered adjuvants (**Figure 1**): influenza virus, rabies virus and the Japanese encephalitis virus are enveloped viruses, while poliovirus is an icosahedral virus without lipid membranes. Therefore, both forms of virus structures, either with or without lipid bilayer membranes, can elicit potent and durable IgG for protection in humans. However, the mobility of viral surface Ags in inactivated enveloped viruses is most likely constrained due to their interactions with viral structural proteins underneath the lipid bilayer and potential crosslinking with other proteins mediated by formaldehyde. The immunogenicity of the SVLS platform in humans and comparison with other rigid platforms remain open questions to be addressed in the future.

## Conclusions

Although the cellular basis for long-term Ab responses has been well established in immunology (16, 240, 241), the molecular compositions of an immunogen that can elicit durable IgG responses remain less understood. The ability of SVLS to induce lifelong Ag-specific IgG in mice from a single injection of Ag at submicrogram doses (162) has important implications for the mechanisms of long-lasting IgG. A striking revelation from the current human vaccine literature review is that engineered adjuvants such as alum are not essential for a long-lasting IgG response even for inactivated virus vaccines. This is important for understanding the molecular basis of viral immunogenicity. We propose that a virus-like structure — devoid of any engineered adjuvants — is sufficient to mediate a long-lasting IgG response in both mice and humans. This structure includes two essential features: (1) the oriented display of viral surface protein Ags on a virus-sized scaffold, and (2) iNAs with native phosphodiester backbones. Clarifying these two tiers of molecular features helps our understanding of viral immunogenicity, which would enable the rational design of next-generation agents to elicit long-lasting IgG for either prophylactic or therapeutic applications.
